# Fetal eye movements in response to a visual stimulus

**DOI:** 10.1002/brb3.1676

**Published:** 2020-07-01

**Authors:** Tim Donovan, Kirsty Dunn, Amy Penman, Robert J. Young, Vincent M. Reid

**Affiliations:** ^1^ Medical Sciences University of Cumbria Lancaster UK; ^2^ Department of Psychology Lancaster University Lancaster UK; ^3^ Department of Physics Lancaster University Lancaster UK

**Keywords:** eye movements, fetal behavior, fetus, prenatal, ultrasound

## Abstract

**Introduction:**

In 2D ultrasound, the lens of the fetal eye can be distinguished as white circles within the hypoechoic eyeball, and eye movements can be visualized from about 15 weeks' gestation. It has been shown that from 31 weeks gestational age the fetal sensory system is capable of directed vision if enough light is available.

**Methods:**

We have developed a light source for delivering visual stimuli to be seen by the fetal eye, using laser dot diodes emitting at 650 nm. The 2D component of 94 fetal ultrasound scans (mean gestational age 240 days), where the light stimulus was presented, was coded to determine whether the eyes moved in response to the stimuli independent of any head movement.

**Results:**

The light stimulus significantly provoked head and eye movements, but after the light was withdrawn the head stopped moving, yet the eyes continued to move.

**Conclusion:**

This provides evidence for visual attention mechanisms that can be controlled through eye movements that are independent of head movements prior to birth.

## Graphical abstract

We have developed a light source for delivering visual stimuli to be seen by the fetal eye, and the 2D component of 94 fetal scans was coded to determine whether the eyes moved in response to the stimuli independent of any head movement. The light stimulus significantly provoked head and eye movements, but after the light was withdrawn the head stopped moving, yet the eyes continued to move. This provides evidence for visual attention mechanisms that can be controlled through eye movements that are independent of head movements prior to birth.

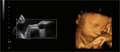

## 1 INTRODUCTION

The investigation of how the human fetus engages with the prenatal sensory, and social and cognitive world is at the core of an intrinsic disconnection in the social developmental sciences. Many have interpreted the demonstration of sensitivity to social and cognitive stimuli in neonates as support for innate origins of understanding or of rapid imprinting of a capacity at birth (DeCasper & Spence, 1986; Morton & Johnson, 1991). With this account, innate interpretations of neonatal capacities discount any effects of prenatal experience on development. Until the 1980s, the prenatal period was viewed as a “developmental limbo” (Hopkins & Johnson, 2005). Only after this period did the experimental study of developmental psychology begin to address the potential for prenatal origins of developmental capacities. Inherent barriers to delivering stimuli to the fetus in utero, especially in the visual domain, have hindered progress (Dunn, Reissland, & Reid, 2015). Some have attempted to overcome these issues by either measuring brain and heart rate responses to auditory stimuli and simple visual stimulations such as flashes of light (e.g., Eswaran, Lowery, Wilson, Murphy, & Preissl, 2005; Weikum, Oberlander, Hensch, & Werker, 2012; Zimmer et al., 1993) or measuring behavior at birth in response to auditory stimuli that have been first presented during the prenatal period (e.g., DeCasper & Spence, 1986). These pioneering studies have provided strong evidence for the development of neonatal capacities in utero; yet, there is a vast literature dedicated to neonatal behavioral responses to visual patterns and sounds that does not include comparable prenatal data. Consequently, we cannot fully understand the connection between, or the transition across, pre‐, neo‐, and postnatal development. As such, the trajectory of early developmental capacities has not been mapped and our understanding of early human development is compartmentalized into specific developmental stages.

In a previously published study (Reid et al., 2017), we have demonstrated that the 34‐week fetus turns the head to follow patterned visual stimuli projected through the uterine wall. Further, a preference was found for the fetus to follow a top‐heavy rather than inverted (bottom‐heavy) shape. This indicates that visual processing of stimuli is occurring in utero and may well be shaping perceptual preferences and capacities. The primary aim of that study was to record head movements using 4D ultrasound to view the face and observe head movements following the presentation of light conveying perceptual content. Fetal head movement is a gross motor movement which is overt and distinct. As such, it is relatively straightforward to code from the ultrasound recordings. When using this measure, however, fetal attention toward stimuli using measures of eye movement will only be intermittently detected.

Most studies on fetal eye movements are over 20 years old and were conducted once the efficacy of real‐time ultrasound in the imaging of the fetal orbit in utero had been demonstrated (e.g., Horimoto, Hepper, Shahidullah, & Koyanagi, 1993). The aim of these studies was generally to qualitatively classify fetal eye movements (Birnholz, 1981) and their relevance to neurological abnormalities. There are very few studies looking at the fetal response to light stimulation and those that have been conducted emphasize their relevance to clinical conditions (Kiuchi, Nagata, Ikeno, & Terakawa, 2000). However, limitations in image quality at 34 weeks gestational age can make the detection of eye movements difficult, and, for the majority of recordings, it is not possible to clearly observe eye movements as a separate phenomenon to head movements in 4D. In 2D ultrasound, however, the lens of the eye can be distinguished as white circles within the hypoechoic eyeball and eye movements can be relatively easily observed (Inoue et al., 1986). As the 4D image is produced by selecting an ideal 2D image within the region of interest, then 2D data are available for review when acquiring 4D ultrasound. This potentially allows for the quantification of eye movements in the human fetus in response to light. As these eye movements would be elicited by a stimulus, it could offer substantial insight into fetal development in utero that would otherwise not be possible were observational techniques to be employed.

Oculomotor control is likely dependent on the extraocular muscles, cranial nerves, and brainstem nuclei which develop at 7–9 weeks (Joseph, 2000). The first eye movements can be seen on 2D ultrasound at 14 weeks gestational age (Horimoto et al., 1993). At 26 weeks (post conception), the fetus has eyelids partially open, by 28 weeks the eyes are wide open and at 31 weeks the pupils can constrict, dilate, and detect light (Kiuchi et al., 2000; Moore, Persaud, & Torchia, 2011). All of the initial eye movements in early fetal development are likely to be reflexive, and rapid fetal eye movements may however be potentially representative of rapid eye movement (REM) sleep. Fetal eye movement activity has been recorded by ultrasound (Okawa et al., 2017) supporting the marked preponderance of REM sleep in the last trimester of pregnancy (Hobson, 2009). By 31 weeks, the fetal visual system is functional (Eswaran et al., 2005). Fetal fMRI studies have provided some insight into this by demonstrating that fetuses react to a constant intensity light source diffused through the maternal abdomen by increased activity in the frontal cortex (Fulford et al., 2003). A resting state fMRI study (36 weeks gestation) has linked spontaneous fetal eye movements with the corresponding functional networks for vision—thereby suggesting that the brain is being prepared for the processing of visual patterns (Schöpf et al., 2014). It has also been suggested that early visuomotor development is primarily driven by a gestational clock rather than early visual experience, though this is based on studies on preterm infants (>33 weeks gestation) where the additional visual experience does not appear to influence development of visual acuity or binocular vision (Weinacht, Kind, Mönting, & Gottlob, 1999). Premature infants do show visual attention and fixation from 30 to 32 weeks, and from 34 weeks newborns can perform ocular motility, object fixation, and detection and tracking of a moving target (Ricci et al., 2010).

Consideration of the fetal environment is important, and although we have a limited understanding of the effect of the fetal physical environment on the development of the fetus there must be a relationship between the fetuses' developing sensory abilities and the uterine environment (André, Henry, Lemasson, Hausberger, & Durier, 2018). It is certain that fetuses do develop in conditions that allow for visual experience before birth (Del Giudice, 2011). There is some continuity between fetal and neonatal behavior, as demonstrated by many observational studies (Almli, Ball, & Wheeler, 2001). In addition, there are no movements in fetuses which are not present in neonates (Stanojevic & Kurjak, 2008). The newborn is socially prepared to recognize human faces at birth, makes eye contact with others, and also responds to biological motion when contrasted with other forms of motion (Pitti, Kuniyoshi, Quoy, & Gaussier, 2013). It has been proposed that birth is not a fundamental shift in developmental capacities (DiPietro, Costigan, & Vogeltine, 2015), with the consequence that these information processing abilities may well be present from 31 weeks gestation. Fetal eye movements may therefore not just be reflexive or random but associated with a history of experience with the environment.

## 2 RESEARCH QUESTION

Can fetal eye movements be observed as a separate phenomenon to head movements when using the 2D component of 4D ultrasound? In the event that fetal eye movements are not spontaneous in nature in utero but responsive to visual stimuli, significantly more eye movements should be found in response to light than in baseline periods.

## 3 MATERIALS AND METHODS

### 3.1 Subjects

One hundred and twenty‐six participants were recruited; however, 32 were excluded due to no image data obtained or corrupt files (*n* = 4), and this left 94 singleton fetuses of gestational age between 33 and 36 weeks (*M* = 239 days *SD* ± 4.7). All participants had a routine pregnancy with no known complications, and at the start of pregnancy a BMI of 30 or less. A higher BMI could cause image quality issues and/or problems with transmission of the light stimulus through the abdominal wall.

All pregnant women participating received written information prior to agreeing to take part in the study and gave informed written consent before participation. This study was approved by the NHS Health Research Authority National Research Ethics Service, the Lancaster University Research Ethics Committee, and the University of Cumbria Ethics Committee.

### 3.2 Image acquisition

Ultrasound scans were performed at Blackpool Victoria Hospital using a GE Healthcare Voluson E8 Expert BT13 advanced 4D HD live ultrasound scanner and 4D probe, model RM66, and at the University of Cumbria Medical Imaging Unit, where a GE Healthcare Voluson iBT07 4D live ultrasound scanner and 4D probe, model RAB4‐8‐RS, was used. The scans were recorded and saved to an external hard drive for off‐line analysis. All scanning was undertaken by appropriately qualified and experienced sonographers. Initially, routine fetal biometry measurements were collected to demonstrate normal growth and no anomalies. An additional measurement of the maternal abdominal thickness (Mean 30.5 mm, *SD* 12.8 mm, range 2.5–82.7 mm), from maternal skin to the uterine wall, was recorded to determine the optical power of the light stimulus used in the stimulus presentation to ensure each fetus was exposed to a constant level of light. The images were acquired with the goal of 4D images, but the data also included the capture of a simultaneous 2D image.

### 3.3 Stimulus

The light source was a custom‐made semiconductor laser torch with an array of dots, emitting light at 650 nm. Depending on the mother's abdominal thickness, the light was calibrated at output optical powers of 0.5, 1, or 5 mW for thickness (*t*) below 1.5 cm, between 1.5 and 3 cm and above 3 cm. This ensured that a consistent level of light was delivered to the fetus irrespective of variations in maternal tissue thickness. Intrauterine illuminance (LI) was calculated using the equation:

$$L_{I}=L_{E}10^{-\left(0.0942+t\frac{0.032+0.058r}{1+r}\right)}$$

modified from Del Giudice (2011) to remove the clothing factor. LE, the external illuminance, was calculated using the output power of the light source, assuming a projected spot size diameter of 10 mm for a maternal tissue thickness of 30 mm, and correcting for the source wavelength of 650 nm, based on values in Stockman, Jägle, Pirzer, and Sharpe (2008). An approximate muscle to fat ratio (*r*) of 2 was used, as in Del Giudice (2011).

The light stimuli are all within the range of the fetal visual system, and a significantly lower level of luminance that the fetus may be exposed to on a bright day (Del Giudice, 2011).

### 3.4 Procedure

The position of the fetus was determined while in 2D mode to ensure the light was presented toward the fetal head, to the side of the fetal face so that the light would be in the peripheral visual field of the fetus. The light stimulus was then moved across the abdomen at a constant rate of 1 cm/s for 5 s. This process was repeated 10 times and was then followed by a period of 3 min constant light (counterbalanced for order). There are consequently three conditions; (a) “Before,” which is a 3 min baseline period immediately before any light was presented, (b) “During,” which is the 3 min of constant light and 50 s of intermittent light presentation with the light moved over the abdomen, and (c) “After,” which is an accumulative period of approximately 4 min after the light stimulus has been removed including the time period between intermittent light presentations.

### 3.5 Data coding

The data presented here are a reanalysis of the dataset consisting of 39 studies presented in Reid et al. (2017) with 55 fetuses additionally included now that the focus is on image quality related to visualization of the lens in 2D rather than the face in 4D. Each study was recorded and videos were analyzed using Windows media player. The number of head and eye movements was recorded for each condition.

**FIGURE 1:**
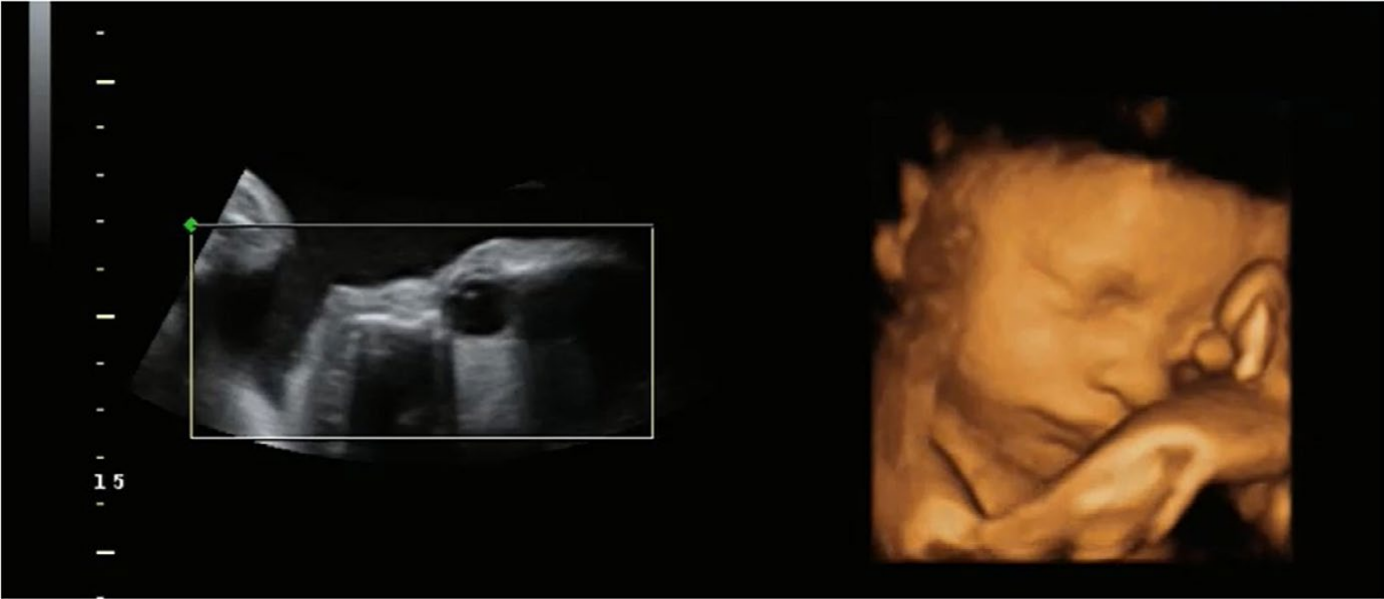
The acoustic reflection from the lens of the eye can be seen in the 2D image on the left, with the corresponding 4D image on the right

Head movements were coded when the center position of the head moved either up or down or left or right and then returned to the starting head position. Eye movements were coded when the lens (the bright white dot on the 2D image (see Figure 1) moved a sufficient amount in any direction around the eyeball when the ultrasound probe was stationary. Head and eye movements were coded before, during, and after light stimulus presentation.

Twenty datasets were coded by two observers, and Pearson's correlation was performed to determine interrater agreement on head and eye movements with a positive correlation between coders for head movements (Pearson's *r* (20) = .583, *p* = .018) and for eye movements (Pearson's *r* (20) = .758, *p* = .001).

## 4 RESULTS

See Table 1.

**TABLE 1:** Mean number of movements per condition

	Head movement			Eye movement		
	Number	Mean	*SE*	Number	Mean	*SE*
Before stimuli	53	0.6	0.09	17	0.2	0.05
During stimuli	193	2.1	0.19	66	0.7	0.12
After stimuli	78	0.8	0.13	60	0.6	0.08

Note

A Wilcoxon signed‐rank test indicated significantly more head movements during light presentation than before (*Z* = −6.320, *p* < .001) and after (*Z* = −5.712, *p* < .001). In addition, significantly more eye movements were recorded during light presentation than before (*Z* = −3.716, *p* < .001) and significantly more eye movements were recorded after light presentation than before (*Z* = −4.155, *p* < .001). No further comparisons were found to be significant.

## 5 DISCUSSION

A significantly greater number of eye movements were found during and after stimulus presentation than before presentation. The number of eye movements during and after stimulus presentation was similar, whereas there were no significant differences between head movements before and after, but a significant difference between head movements during and before, as well as during and after (both *p* < .001). Thus, the light stimulus provoked head and eye movements, but after the light was withdrawn the head stopped moving—presumably because there was no stimulus to orient toward—yet the eyes continued to move. Continued eye movements may be due to the result of residual activation of the visual system or residual heightened arousal leading to continued lens movement. Yet an alternative interpretation of these data is that the fetus was detecting a change in the environment and responding to the light stimulus utilizing eye movements. This interpretation of the data suggests fetal awareness of the environment. A view present in the literature is that the fetus is actively maintained asleep during uterine life by endogenous substances such as intrauterine endocrine neuroinhibitors, that is, the fetus lacks “awareness” (Lagercrantz, 2016; Padilla & Lagercrantz, 2020). It has also been argued there is no evidence that the fetus lacks awareness or exists in a different conscious state (Platt, 2011). Separate to these issues, observational studies have classified fetal behavior after 32 weeks gestation using observational data (Nijhuis, 2003), resulting in four states (quiet sleep, active sleep, quiet awake, active awake) with the fetus seeming to be predominantly in “active sleep” (Brändle et al., 2015; Nijhuis, 2003; Pillai & James, 1990). A change in fetal state in response to a stimulus would suggest fetal capacity to detect change in the environment. This was explored by Kiuchi et al. (2000) using a visual stimulus (photographic flash) and vibroacoustic stimulation. They found a high proportion (82%) of a positive response, as determined by an actocardiograph and ultrasonography, to the light stimulus when the fetus was in “active sleep” and “quiet awake.” Response to such stimuli may however include nonwaking cortical arousal rather than an awake state or a transition between sleep states (Mellor, Diesch, Gunn, & Bennet, 2005).

It is important to acknowledge that as the fetus develops, a large proportion of time is spent in REM sleep (Mirmiran, Maas, & Ariagno, 2003). It is difficult to determine how much time is spent in REM sleep, but the development of non‐REM and REM eye movements has been assessed quantitatively using 2D ultrasound (Inoue et al., 1986), based on 1 min continuous observations. These demonstrated a rise in REM with gestational age. Unlike observational studies, the present study is comparing the systematic response to the onset and offset of externally presented stimuli, with the consequence that any noise from random eye movements are as likely at each time period. Differences in sleep states cannot, therefore, explain the differences seen between conditions in the present study.

Shifts of visual attention are guided reflexively or exogenously. Observational studies using 4D ultrasound, which are important in gaining an insight into fetal behavior, have indicated a level of anticipatory awareness in the fetus. Myowa‐Yamakoshi and Takeshita (2006) observed hand and arm movements between 19 and 35 weeks of gestation finding fetuses opened their mouths before hands came into contact with the face. Reissland, Francis, Aydin, Mason, and Schaal (2014) further investigated anticipatory touch behavior finding qualitative differences in mouth‐touch behavior from 24 to 36 weeks gestation. A developmental shift was reported from mouth openings following a mouth touch (reactive) in earlier weeks to mouth openings before mouth touch (anticipatory) in later weeks. Reid et al. (2017) showed that at 34 weeks gestation the fetus is perceiving information in the womb which, when processed, will elicit an overt orienting response. This interpretation explains the eye and head movements during the stimulus presentation as reported in the present study. Our study also demonstrates that shifts of visual attention can be generated endogenously in the fetus. Amso and Johnson (2006) ask the question “at what stage does an infant become an active participant in their own perceptual development?” Our data indicate that the fetus has this capacity before birth.

### 5.1 Limitations

In this study, 2D data were generally acquired in the sagittal plane before switching to 4D, whereas the coronal or axial planes are often better suited for imaging of the orbits. In future studies, recording in these more appropriate planes may well increase observer reliability.

In our study, we did not assess fetal state as a way of differentiating those that were asleep or awake. Despite this, previous studies have suggested that the fetus is predominantly in “active sleep” and yet in the present study, presentation of stimuli still elicited a positive response. It is possible that the boundary between “active sleep” and “quiet awake” is not as clearly delineated as previously thought and the classification of behavioral states overlap due to a lack of behavioral measures to classify states. Undoubtedly, there will be proportion of our data where the fetus is in a sleep state. Despite this, significant differences are found without filtering the data.

## 6 CONCLUSION

Even when taking these limitations into account, these results have important implications for developmental theories surrounding the origins of newborn infant visual capacities. As experimental techniques using prenatal samples are improving, a consistent pattern is emerging that points toward prenatal environmental influence on the developing visual system. This casts doubt on the likelihood of “nativist only” origins of development.

## CONFLICT OF INTEREST

The authors declare no competing interests in the subject matter or materials discussed in this manuscript.

## AUTHOR CONTRIBUTIONS

T.D. and K.D. wrote the manuscript. V.M.R., K.D., and T.D. conceived the idea of the presented work. K.D. collected the data. A.P., K.D., and T.D. analyzed the data. K.D., R.J.Y., and V.M.R. contributed to methodology. All authors reviewed and contributed to the final manuscript.

## Data Availability

The data that support the findings of this study are available on request from the corresponding author. The data are not publicly available due to privacy or ethical restrictions.

## References

[brb31676-bib-0001] Almli, C. R. , Ball, R. H. , & Wheeler, M. E. (2001). Human fetal and neonatal movement patterns: Gender differences and fetal‐to‐neonatal continuity. Developmental Psychobiology, 38(4), 252–273. 10.1002/dev.1019 11319731

[brb31676-bib-0002] Amso, D. , & Johnson, S. P. (2006). Learning by selection: Visual search and object perception in young infants. Developmental Psychology, 42(6), 1236–1245. 10.1037/0012-1649.42.6.1236 17087555

[brb31676-bib-0003] André, V. , Henry, S. , Lemasson, A. , Hausberger, M. , & Durier, V. (2018). The human newborn's umwelt: Unexplored pathways and perspectives. Psychonomic Bulletin & Review, 25(1), 350–369. 10.3758/s13423-017-1293-9 28462504

[brb31676-bib-0004] Birnholz, J. C. (1981). The development of human fetal eye movement patterns. Science, 213(4508), 679–681. 10.1126/science.7256272 7256272

[brb31676-bib-0005] Brändle, J. , Preissl, H. , Draganova, R. , Ortiz, E. , Kagan, K. O. , Abele, H. , … Kiefer‐Schmidt, I. (2015). Heart rate variability parameters and fetal movement complement fetal behavioral states detection via magnetography to monitor neurovegetative development. Frontiers in Human Neuroscience, 9, 147 10.3389/fnhum.2015.00147 25904855 PMC4388008

[brb31676-bib-0006] DeCasper, A. J. , & Spence, M. J. (1986). Prenatal maternal speech influences newborns' perception of speech sounds. Infant Behavior and Development, 9(2), 133–150. 10.1016/0163-6383(86)90025-1

[brb31676-bib-0007] Del Giudice, M. (2011). Alone in the dark? Modeling the conditions for visual experience in human fetuses. Developmental Psychobiology, 53(2), 214–219. 10.1002/dev.20506 21298635

[brb31676-bib-0008] DiPietro, J. A. , Costigan, K. A. , & Vogeltine, K. M. (2015). Studies in fetal behaviour: Revisited, renewed, and reimagined. Monographs for the Society for Research in Child Development, 80(3), 1–94. 10.1111/mono.v80.3 PMC4835043 26303396

[brb31676-bib-0009] Dunn, K. , Reissland, N. , & Reid, V. M. (2015). The functional foetal brain: A systematic preview of methodological factors in reporting foetal visual and auditory capacity. Developmental Cognitive Neuroscience, 13, 43–52. 10.1016/j.dcn.2015.04.002 25967364 PMC6990098

[brb31676-bib-0010] Eswaran, H. , Lowery, C. L. , Wilson, J. D. , Murphy, P. , & Preissl, H. (2005). Fetal magnetoencephalography—A multimodal approach. Developmental Brain Research, 154(1), 57–62. 10.1016/j.devbrainres.2004.10.003 15617755

[brb31676-bib-0011] Fulford, J. , Vadeyar, S. H. , Dodampahala, S. H. , Moore, R. J. , Young, P. , Baker, P. N. , … Gowland, P. A. (2003). Fetal brain activity in response to a visual stimulus. Human Brain Mapping, 20(4), 239–245. 10.1002/hbm.10139 14673807 PMC6871889

[brb31676-bib-0012] Hobson, J. A. (2009). REM sleep and dreaming: towards a theory of protoconsciousness. Nature Reviews Neuroscience, 10(11), 803–813. 19794431 10.1038/nrn2716

[brb31676-bib-0013] Hopkins, B. , & Johnson, S. P. (2005). Prenatal development of postnatal functions. Westport, CT: Greenwood Publishing Group.

[brb31676-bib-0014] Horimoto, N. , Hepper, P. G. , Shahidullah, S. , & Koyanagi, T. (1993). Fetal eye movements. Ultrasound in Obstetrics & Gynecology, 3(5), 362–369. 10.1046/j.1469-0705.1993.03050362.x 12797264

[brb31676-bib-0015] Inoue, M. , Koyanagi, T. , Nakahara, H. , Hara, K. , Hori, E. , & Nakano, H. (1986). Functional development of human eye movement in utero assessed quantitatively with real‐time ultrasound. American Journal of Obstetrics and Gynecology, 155(1), 170–174. 10.1016/0002-9378(86)90105-5 3524238

[brb31676-bib-0016] Joseph, R. (2000). Fetal brain behavior and cognitive development. Developmental Review, 20(1), 81–98.

[brb31676-bib-0017] Kiuchi, M. , Nagata, N. , Ikeno, S. , & Terakawa, N. (2000). The relationship between the response to external light stimulation and behavioral states in the human fetus: How it differs from vibroacoustic stimulation. Early Human Development, 58(2), 153–165. 10.1016/S0378-3782(00)00074-8 10854802

[brb31676-bib-0018] Lagercrantz, H. (2016). Infant brain development. Berlin, Germany: Springer.

[brb31676-bib-0019] Mellor, D. J. , Diesch, T. J. , Gunn, A. J. , & Bennet, L. (2005). The importance of ‘awareness’ for understanding fetal pain. Brain Research Reviews, 49(3), 455–471. 10.1016/j.brainresrev.2005.01.006 16269314

[brb31676-bib-0020] Mirmiran, M. , Maas, Y. G. , & Ariagno, R. L. (2003). Development of fetal and neonatal sleep and circadian rhythms. Sleep Medicine Reviews, 7(4), 321–334. 10.1053/smrv.2002.0243 14505599

[brb31676-bib-0021] Moore, K. L. , Persaud, T. V. N. , & Torchia, M. G. (2011). The developing human e‐book. Amsterdam, the Netherlands: Elsevier Health Sciences.

[brb31676-bib-0022] Morton, J. , & Johnson, M. H. (1991). CONSPEC and CONLERN: A two‐process theory of infant face recognition. Psychological Review, 98(2), 164 10.1037/0033-295X.98.2.164 2047512

[brb31676-bib-0023] Myowa‐Yamakoshi, M. , & Takeshita, H. (2006). Do human fetuses anticipate self‐oriented actions? A study by four‐dimensional (4D) ultrasonography. Infancy, 10(3), 289–301. 10.1207/s15327078in1003_5

[brb31676-bib-0024] Nijhuis, J. G. (2003). Fetal behavior. Neurobiology of Aging, 24, S41–S46. 10.1016/S0197-4580(03)00054-X 12829106

[brb31676-bib-0025] Okawa, H. , Morokuma, S. , Maehara, K. , Arata, A. , Ohmura, Y. , Horinouchi, T. , … Kato, K. (2017). Eye movement activity in normal human fetuses between 24 and 39 weeks of gestation. PLoS One, 12(7), e0178722. 28700709 10.1371/journal.pone.0178722 PMC5507482

[brb31676-bib-0026] Padilla, N. , & Lagercrantz, H. (2020). Making of the mind. Acta Paediatrica, 109, 883–892. 10.1111/apa.15167 31922622

[brb31676-bib-0027] Pillai, M. , & James, D. (1990). Behavioural states in normal mature human fetuses. Archives of disease in childhood, 65(1 Spec No), 39–43. 2306133 10.1136/adc.65.1_spec_no.39 PMC1590168

[brb31676-bib-0028] Pitti, A. , Kuniyoshi, Y. , Quoy, M. , & Gaussier, P. (2013). Modeling the Minimal Newborn’s Intersubjective Mind: The Visuotopic‐Somatotopic Alignment Hypothesis in the Superior Colliculus. PLOS ONE, 8(7), e69474 10.1371/journal.pone.0069474. 23922718 PMC3724856

[brb31676-bib-0029] Platt, M. W. (2011). Fetal awareness and fetal pain: The Emperor's new clothes. Archives of Disease in Childhood ‐ Fetal and Neonatal Edition, 96(4), F236–F237. 10.1136/adc.2010.195966 21292851

[brb31676-bib-0030] Reid, V. M. , Dunn, K. , Young, R. J. , Amu, J. , Donovan, T. , & Reissland, N. (2017). The human fetus preferentially engages with face‐like visual stimuli. Current Biology, 27(12), 1825–1828.e3. 10.1016/j.cub.2017.05.044 28602654

[brb31676-bib-0031] Reissland, N. , Francis, B. , Aydin, E. , Mason, J. , & Schaal, B. (2014). The development of anticipation in the fetus: A longitudinal account of human fetal mouth movements in reaction to and anticipation of touch. Developmental Psychobiology, 56(5), 955–963. 10.1002/dev.21172 24752616

[brb31676-bib-0032] Ricci, D. , Romeo, D. M. , Serrao, F. , Gallini, F. , Leone, D. , Longo, M. , … Mercuri, E. (2010). Early assessment of visual function in preterm infants: How early is early? Early Human Development, 86(1), 29–33. 10.1016/j.earlhumdev.2009.11.004 20056358

[brb31676-bib-0033] Schöpf, V. , Schlegl, T. , Jakab, A. , Kasprian, G. , Woitek, R. , Prayer, D. , & Langs, G. (2014). The relationship between eye movement and vision develops before birth. Frontiers in Human Neuroscience, 8, 775 10.3389/fnhum.2014.00775 25324764 PMC4183095

[brb31676-bib-0034] Stanojevic, M. , & Kurjak, A. (2008). Continuity between Fetal and Neonatal Neurobehavior. Donald School Journal of Ultrasound in Obstetrics & Gynecology, 2, 64–75. 10.5005/jp-journals-10009-1066

[brb31676-bib-0035] Stockman, A. , Jägle, H. , Pirzer, M. , & Sharpe, L. T. (2008). The dependence of luminous efficiency on chromatic adaptation. Journal of Vision, 8(16), 1–1. 10.1167/8.16.1 19146268

[brb31676-bib-0036] Weikum, W. M. , Oberlander, T. F. , Hensch, T. K. , & Werker, J. F. (2012). Prenatal exposure to antidepressants and depressed maternal mood alter trajectory of infant speech perception. Proceedings of the National Academy of Sciences of the United States of America, 109(Supplement 2), 17221–17227. 10.1073/pnas.1121263109 23045665 PMC3477387

[brb31676-bib-0037] Weinacht, S. , Kind, C. , Mönting, J. S. , & Gottlob, I. (1999). Visual development in preterm and full‐term infants: A prospective masked study. Investigative Ophthalmology & Visual Science, 40(2), 346–353. 9950592

[brb31676-bib-0038] Zimmer, E. Z. , Fifer, W. P. , Kim, Y.‐I. , Rey, H. R. , Chao, C. R. , & Myers, M. M. (1993). Response of the premature fetus to stimulation by speech sounds. Early Human Development, 33(3), 207–215. 10.1016/0378-3782(93)90147-M 8223316

